# Genome-wide functions of PML–RAR*α* in acute promyelocytic leukaemia

**DOI:** 10.1038/sj.bjc.6606095

**Published:** 2011-01-18

**Authors:** S Saeed, C Logie, H G Stunnenberg, J H A Martens

**Affiliations:** 1 Department of Molecular Biology, Faculty of Science, Nijmegen Centre for Molecular Life Sciences, Radboud University, 6500 HB Nijmegen, The Netherlands

**Keywords:** PML-RAR*α*, RXR, PU.1 (SPI1), epigenome

## Abstract

PML—RAR (retinoic acid receptor)*α* is the hallmark protein of acute promyelocytic leukaemia, a highly malignant subtype of acute myeloid leukaemia that accounts for approximately 10% of all AML cases. Recently, several studies have been set out to obtain a comprehensive genome-wide view of the molecular actions of this chimeric protein. In this review, we highlight the new insights that arose from these studies, in particular focussing on newly identified PML–RAR*α* target genes, its interplay with RXR and deregulation of epigenetic modifications.

Acute promyelocytic leukaemia (APL) is a distinctive subtype of acute myeloid leukaemia (AML) that accounts for approximately 10% of all AML cases (Jing, 2004). The disease represents a highly malignant form of leukaemia with high bleeding tendency and a fatal course of only few weeks (Wang and Chen, 2008). The main diagnostic feature of APL is an aberrant chromosomal translocation that juxtaposes the PML gene on chromosome 15 and the retinoic acid receptor (RAR)*α* gene on chromosome 17 (Kakizuka *et al*, 1991). The resultant chimeric protein, which is found in over 95% of human APLs (Di Croce, 2005), retains the DNA-binding and ligand-binding domains of RAR*α* and the multimerisation domain of PML. In normal cells PML is a main constituent of nuclear bodies, which are matrix-associated multiprotein-containing domains involved in various biological functions like DNA-damage response and microorganism resistance through regulation of a wide range of proteins, among which are various transcription factors (Lallemand-Breitenbach and de The, 2010). In contrast, in APL, the expression of PML–RAR*α* disrupts the localisation of the wild-type PML from nuclear bodies to numerous micro speckles (Brown *et al*, 2009) and induces a maturation block at the promyelocytic level (Wang and Chen, 2008). All-*trans* retinoic acid (ATRA) and arsenic trioxide (ATO) are the two most important drugs in clinical use for the treatment of early-diagnosed APL. Both ATRA and ATO degrade the PML–RAR*α* fusion protein by acting on the RAR*α* and PML moieties, respectively. ATRA mainly degrades the protein through proteosome-mediated pathways (Zhu *et al*, 1999) and caspases (Nervi *et al*, 1998), while ATO-induced degradation is initiated through sumoylation of the PML moiety. Both treatments ultimately lead to restoration of PML nuclear bodies (Lallemand-Breitenbach *et al*, 2008; Zhang *et al*, 2010), but whether this is important for curing the disease is an open question.

Various mechanisms have been proposed for PML–RAR*α* functioning. It has been suggested that PML–RAR*α* can form homodimers without RXR (Minucci *et al*, 2000) or that it forms PML–RAR*α* oligomers that heterodimerise with RXR (Perez *et al*, 1993; Jansen *et al*, 1995). In addition, it has been suggested that during transformation PML–RAR*α* induces a multitude of alterations in the chromatin architecture. These alterations are achieved through the recruitment of various epigenetic-modifying factors, like histone deacetylase complexes such as SMRT (Lin *et al*, 1998) and N-CoR (Grignani *et al*, 1998), and DNA methyltransferases (Di Croce *et al*, 2002). In addition, recent evidence suggests co-recruitment of the histone methyltransferases SUV39H1 and polycomb repressor complexes, which dictate the epigenetic state of H3K9 (Carbone *et al*, 2006) and H3K27 (Villa *et al*, 2007), respectively. Unfortunately, most of these studies showed epigenetic alterations only around a limited set of binding regions, in most cases the RAR*β* promoter. This was largely due to unavailability of the genome-wide PML–RAR*α* target site repertoire. However, the recent advances in high-throughput tools have, for the first time, made it possible to look at the genome-wide actions of PML–RAR*α* and different epigenetic marks associated with its binding. Indeed, two recent studies have provided a more global picture of PML–RAR*α* functioning by identifying binding regions of PML–RAR*α,* using state-of-the-art ChIP-seq and ChIP-on-chip technologies. Importantly, the new technologies even allowed extension from the commonly used model cell lines to primary APL blasts, highlighting the new opportunities that have now become available.

In this review we will focus on the various aspects of PML–RAR*α* functioning with respect to its genome-wide binding spectrum, its interplay with RXR and its regulation of various epigenetic modifications. In addition, we will discuss some of the newly identified target genes and target pathways of PML–RAR*α*.

## Altered RAR signalling in APL cells

All-*trans* retinoic acid belongs to the group of vitamin A-derived substances and binds three major RARs, RAR*α*, *β* and *γ*. The full execution of the ATRA signalling pathway operates by inducibly controlling the expression of the genes that have a direct repeat with spacing 2 or 5 (DR2 or DR5) in their promoter (de The *et al*, 1991). Retinoid signalling has a key role in various developmental and differentiation processes. According to the classical model, RAR and RXR, another nuclear receptor, heterodimerise under non-ATRA conditions, recruit co-repressors and silence target gene expression. In this model, RAR binding to ATRA results in a conformational change in the RXR–RAR heterodimer, allowing recruitment of gene-activating complexes and resultant gene expression. In APL, PML–RAR*α* is thought to behave functionally as an altered RAR*α* that has lost the potential to respond to fluctuations in physiological ATRA concentration, and as a result acts as a constitutive transcriptional repressor for DR2- or DR5-containing genes. Strikingly, recent studies that analysed the genome-wide PML–RAR*α* binding sites not only identified DR2 and DR5 elements as the primary PML–RAR*α* response elements, but also regions containing DR1, DR3 and DR4 motifs and even more atypical DR motifs with various spacing and orientation were detected (Martens *et al*, 2010), thereby extending *in vivo* previous *in vitro* data (Kamashev *et al*, 2004). This rather diverse repertoire of response elements present at the PML–RAR*α* binding sites underscores the idea of a gain of DNA-binding capacity as an essential feature of PML–RAR*α*-mediated transformation. In addition to this extended binding potential, global binding data of PML–RAR*α* using ChIP-seq revealed PML–RAR*α* binding to the RAR*α*, RAR*β* and RAR*γ* genes itself (Table 1), suggesting that expression of these proteins is directly regulated by PML–RAR*α* (Martens *et al*, 2010). All these data suggest that PML–RAR*α* affects ATRA signalling at multiple levels: first by regulating expression of the genes involved in transmitting the ATRA signals, second through an altered regulation of classical DR2-and DR5-containing RAR/RXR target genes and third through an extension of the (PML-)RAR binding potential towards more degenerate DR-containing regulatory sites.

## PML–RAR*α* heterodimerises with RXR

Although oligomerisation of the RAR*α* fusion proteins has been considered to be a crucial requirement to their oncogenic potential (Minucci *et al*, 2000), several studies support a role of RXR in the PML–RAR*α* transformation process. RXR was already described as part of the PML–RAR*α* oncogenic complex in *in vitro* settings (Kamashev *et al*, 2004). In addition, recent studies highlighted the importance of RXR in PML–RAR*α*-mediated transcriptional repression (Zeisig *et al*, 2007; Zhu *et al*, 2007). These studies showed that impaired RXR binding by PML–RAR*α* mutants impairs APL development in transgenic mice while still retaining the transforming potential *in vitro*. Furthermore, they showed that silencing of RXR by shRNA suppresses the RAR*α* fusion-mediated transformation *in vitro*. These studies were corroborated and extended by the identification of genome-wide association of RXR with PML–RAR*α* (Martens *et al*, 2010). For 99% of PML–RAR*α* binding sites, association of RXR was detected. Together, these studies highlight the importance of RXR in the PML–RAR*α*-mediated transformation process. It may therefore be interesting to also test RXR antagonists (Altucci *et al*, 2007) in this subtype of AML.

## PML–RAR*α* cross talks with other transcription factors

Apart from interacting with RXR, PML–RAR*α* has been suggested to interact with many other proteins. Amongst these are various key haematopoietic regulatory transcription factors such as GATA-2, PU.1 and AP-1 factors. PML–RAR*α* is proposed to be involved in inhibition of AP-1 transcriptional activity in an ATRA-dependent manner. This is illustrated by the observation that PML–RAR*α* interacts with c-Jun and c-Fos and that the repressive effect on AP1 target sites is reversed by ATRA treatment (Doucas *et al*, 1993). There is also evidence of a physical association of GATA2 with PML–RAR*α*. This interaction is suggested to result in enhanced GATA-2-dependent transactivation capacity (Tsuzuki *et al*, 2000). In line with these two studies is the observation that PML–RAR*α* binding was detected not only near several AP1 factors, such as JUNB and JUND, but also near GATA2 (Table 1) (Martens *et al*, 2010), suggesting that these factors are affected both at the level of their expression and in their targeting capacities. In addition, we observed PML–RAR*α* binding at several other key regulators of haematopoiesis such as PU.1 (see below) RUNX1, RUNX3 and GFI1 (Table 1). This extends the observation that PML–RAR*α* regulates the classical targets of the retinoic acid signalling pathway to many other key players in haematopoiesis.

## Binding of PML–RAR*α* with PU.1

PU.1 is a protein that is essential for the haematopoietic differentiation process. PU.1 mutants lead to embryonic lethality at a late gestational stage (Scott *et al*, 1994). Mice that have homozygous disruption of the DNA-binding domain of PU.1 have severe septicaemia and die within 48 h of their birth (McKercher *et al*, 1996). In addition, PU.1 has been shown to be essential for reprogramming B-cells into the myeloid lineage (Xie *et al*, 2004). Already in 2006 it was proposed that PML–RAR*α* interacts with PU.1 and that the action of PU.1 is suppressed upon expression of PML–RAR*α*, thereby resulting in a differentiation block (Mueller *et al*, 2006). In these studies, ATRA treatment and the ensuing PML–RAR*α* degradation resulted in restoration of PU.1 expression and a release of the differentiation block. These studies already hinted at the molecular interplay of PU.1 with PML–RAR*α*. A recent study based on genome-wide binding of PML–RAR*α* using ChIP-sequencing in a PML–RAR*α* inducible cell model further shed light on the PU.1 and PML–RAR*α* interaction (Wang *et al*, 2010). In this study, more than 84% of the detected PU.1 motifs were found in the close vicinity of variably spaced direct repeats (DRs). Further functional analysis suggested that the binding of PML–RAR*α* to the regions containing both PU.1 and DRs is a prerequisite for subsequent repression of chromatin at PU.1 targeted regions. In addition to these observations, the PU.1 gene was found to be a direct target of PML–RAR*α* (Martens *et al*, 2010). Interestingly, binding of PML–RAR*α* was not detected at the promoter regions but rather in the third intron of the PU.1 gene (Table 1). As this intronic region has also been reported to contain the transcription start site of an antisense transcript that acts as a putative negative regulator of PU.1 expression (Ebralidze *et al*, 2008), these results identify for the first time PML–RAR*α*-mediated regulation of a non-coding transcript.

## PML–RAR*α* affects the epigenome

Multiple studies have suggested that central to oncogenic transformation in APLs is the PML–RAR*α*-induced mis-targeting of the epigenetic machinery, thereby causing a perturbation of the normal epigenetic landscape. Genome-wide binding analysis of PML–RAR*α* (Martens *et al*, 2010) revealed that various enzymes that can set different chromatin modifications are targeted by PML–RAR*α*, including JMJD3 (H3K27me3 demethylation), SETDB1, JMJD1A (H3K9 modifiers), deacetylases like HDAC4 and 9, and genes involved in DNA methylation, such as DNMT3A (Table 1). These findings suggested that PML–RAR*α* expression has the potential to confer a genome-wide alteration in epigenetic make-up. In addition to the direct transcriptional regulation of epigenetic enzymes, different labs have explored the epigenetic marks that are recruited by PML–RAR*α* itself. Histone marks such as H3K27me3 and H3K9me3, as well as DNA methylation, have been proposed to be positively correlated with PML–RAR*α* binding, whereas H3 acetylation was associated with loss of PML–RAR binding. The dynamic changes of DNA methylation, H3K9me3 and H3K27me3 functioning are suggested to be regulated by DNA methyltransferases, histone methyltransferase (SUV39H1) and polycomb repressive complex 2 (PRC2), respectively, and all these proteins have been suggested to interact with the PML–RAR*α* complex in several independent studies (Di Croce *et al*, 2002; Carbone *et al*, 2006; Villa *et al*, 2007). H3 acetylation was the first chromatin modification associated with PML–RAR*α* binding and is a mark that is negatively correlated with PML–RAR*α* binding. Mechanistically, this is thought to be achieved through PML–RAR*α*-mediated recruitment of HDACs (Grignani *et al*, 1998; Lin *et al*, 1998). Indeed, *in vivo* studies could show that there is a significant increase of H3 acetylation upon ATRA-mediated degradation of PML–RAR*α* at the RAR*β* promoter (Villa *et al*, 2007). Still, the limiting factor to this observation and also other studies that investigated epigenetic marks was the number of PML–RAR*α* binding regions addressed and, therefore, the generality of the proposed mechanisms. The recent genome-wide interrogation of APL (Martens *et al*, 2010) allowed for the first time expansion to all binding regions of PML–RAR*α*. This showed that there was a significant regulation of local H3 acetylation at more than 80% of the PML–RAR*α* binding regions, illustrated by the observation that H3 acetylation levels at these sites were significantly elevated upon ATRA-induced PML–RAR*α* degradation. In contrast, changes in other epigenetic marks such as H3K9me3, H3K27me3 and DNA methylation could not be generalised towards all PML–RAR*α* binding sites, as the vast majority of sites did not show a significant change after ATRA treatment. These findings are of significant worth, as they point out the importance of the role of histone deacetylases in maintenance of repressed chromatin architecture at PML–RAR*α* binding sites. This sanctions the idea of making HDACs direct targets for therapeutic treatment of APL by using specific inhibitors for these proteins.

## Outlook

**Figure 1:**
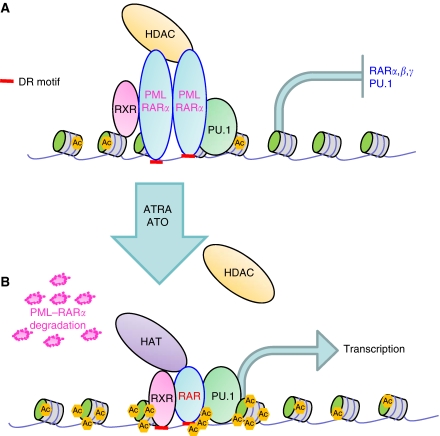
Model of PML–RAR*α* binding. (**A**) PML–RAR*α* in conjunction with RXR and PU.1 binds DR motifs and recruits repressor complexes, resulting in histone hypoacetylation and transcriptional silencing. (**B**) All-*trans* retinoic acid (ATRA) or arsenic trioxide (ATO) mediates degradation of PML–RAR*α*, which is replaced by the RAR*α*/RXR heterodimer, resulting in recruitment of activating complexes and transcriptional activation. HAT, histone acetyltransferase; HDAC, histone deacetylase.

In this review, we focused on two recent studies that describe a global analysis of PML–RAR*α* (Martens *et al*, 2010; Wang *et al*, 2010). Both studies have shown nearly 3000 genome-wide direct targets of PML–RAR*α* by using high-throughput sequencing and array-based technologies, and provided a significant step forward in understanding PML–RAR*α*-mediated leukaemogenesis. Although these studies used independent platforms, several common conclusions were drawn (Figure 1). One of these is the extended binding repertoire of PML–RAR*α* in comparison with the non-fused RAR. Indeed, direct, inverted and everted repeats with various spacing and orientation were detected *in vivo* for PML–RAR*α* binding sites in comparison with the classical DR2 and DR5 motifs that are the hallmarks of heterodimerised RAR*α*/RXR binding regions. In addition to the DR motifs, the discovery of DNA motifs for PU.1 resulted in the finding that PU.1 colocalises with PML–RAR.

The above studies highlighted the fact that PML–RAR*α* not only binds to the promoter regions of target genes but also has a rather diverse repertoire of binding sites. This complex binding spectrum suggests a potential influence on long-range chromosomal interactions by PML–RAR*α*. The wide-ranging molecular alterations induced by PML–RAR*α* are further illustrated by the fact that PML–RAR*α* regulates several key regulators of normal haematopoiesis, such as PU.1, GATA-2, RUNX1 and many others, as well as different pathways such as RAR signalling. In addition, exploration of the epigenetic environment of the APL genome before and after ATRA treatment gave significant insights into PML–RAR*α* chromatin regulation. An inverse correlation of PML–RAR*α* with H3 acetylation was revealed at the PML–RAR*α* binding sites themselves, while the genome-wide epigenetic environment was also significantly remodelled. Still, more in-depth functional studies are needed to provide an answer as to whether histone H3 acetylation levels or other epigenetic markings are crucial in the PML–RAR*α*-induced transformation process. Nevertheless, current data already hint at potential drug targets such as acetyltransferases and deacetylases, as well as RXR for treatment of APL.

Despite the wealth of important insights on PML–RAR*α* functioning provided by the above studies, the question still remains as to what the crucial determinant for PML–RAR*α* binding to a particular region actually is. Is it the underlying motif, interaction with other transcription factors, the chromatin accessibility, or a combination of all three? In order to obtain a better comprehension of normal haematopoiesis and leukaemia, it will be important to address these crucial questions. At the same time, deeper insight is required into the molecular behaviour of other oncofusion proteins that harbour functional properties similar to PML–RAR*α*'s such as AML1-ETO, which is the result of the t(8;21) chromosomal translocation, or the inv (16) translocation that gives rise to the CBF*β*-MYH11 oncofusion protein (Martens and Stunnenberg, 2010). A comparative analysis of the molecular actions of several oncofusion proteins is expected to uncover some of the more general mechanisms that are used by these proteins to transform cells.

## Figures and Tables

**Table 1:** Binding targets of PML–RAR*α* (HG18)

**Gene name**	**Chromosome**	**Start PML–RAR*α* peak**	**End PML–RAR*α* peak**	**Peak location**
*GFI1*	chr1	92714254	92714749	Gene body
*RUNX1*	chr21	35159419	35160205	Gene body
*RUNX3*	chr1	25221279	25222212	Distant
*JUND*	chr19	18263175	18264260	Upstream far
*JUNB*	chr19	12760448	12760879	Upstream near
*GATA2*	chr3	129725052	129725491	Distant
*SETDB1*	chr1	149165218	149165613	Upstream near
*DNMT3A*	chr2	25377769	25378201	Gene body
*JMJD1A*	chr2	86521292	86521853	Upstream near
*HDAC4*	chr2	239913436	239913935	Gene body
*HDAC9*	chr7	18323574	18323895	Distant
*PRMT3*	chr11	20365226	20365660	Upstream near
*SETD8*	chr12	122434062	122434727	Gene body
*PRMT7*	chr16	66947562	66948286	Gene body
*JMJD3*	chr17	7682360	7683526	Upstream near
*DOT1L*	chr19	2119325	2119752	Gene body
*PU.1 (SPI1)*	chr11	47337456	47338471	Gene body
*RARA*	chr17	35762690	35763210	Gene body
*RARB*	chr3	25444370	25444950	Upstream near
*RARG*	chr12	51897334	51897732	Gene body

Abbreviation: RAR, retinoic acid receptor.
